# CBX4 promotes antitumor immunity by suppressing *Pdcd1* expression in T cells

**DOI:** 10.1002/1878-0261.13516

**Published:** 2023-10-09

**Authors:** Liwei Ren, Ziyin Li, Yu Zhou, Jun Zhang, Ziheng Zhao, Zhaofei Wu, Ye Zhao, Yurong Ju, Xuewen Pang, Xiuyuan Sun, Wei Wang, Yu Zhang

**Affiliations:** ^1^ Department of Immunology, School of Basic Medical Sciences, NHC Key Laboratory of Medical Immunology Peking University Beijing China; ^2^ Department of Pharmacology Institute of Materia Medica, Peking Union Medical College, Chinese Academy of Medical Sciences Beijing China; ^3^ Institute of Biological Sciences Jinzhou Medical University China

**Keywords:** antitumor immunity, CBX4, H2AK119ub1, PD‐1, PRC1, T‐cell response

## Abstract

E3 SUMO‐protein ligase CBX4 (CBX4), a key component of polycomb‐repressive complexes 1 (PRC1), has been reported to regulate a variety of genes implicated in tumor growth, metastasis, and angiogenesis. However, its role in T‐cell‐mediated antitumor immunity remains elusive. To shed light on this issue, we generated mice with T‐cell‐specific deletion of *Cbx4*. Tumor growth was increased in the knockout mice. Additionally, their tumor‐infiltrating lymphocytes exhibited impaired tumor necrosis factor‐alpha (TNF‐α) and interferon‐gamma (IFN‐γ) production, with an elevated programmed cell death protein 1 (PD‐1) level. In fact, dysregulated *Pdcd1* expression was observed in all major subsets of peripheral T cells from the knockout mice, which was accompanied by a functional defect in response to T‐cell receptor (TCR) stimulation. In support of a direct link between CBX4 and PD‐1, *Cbx4* overexpression resulted in the downregulation of *Pdcd1* expression. Epigenetic analyses indicated that *Cbx4* deficiency leads to diminished accumulation of inhibitory histone modifications at conserved region (*CR*)‐*C* and *CR‐B* sites of the *Pdcd1* promoter, namely mono‐ubiquitinated histone H2A at lysine 119 (H2AK119ub1) and trimethylated histone H3 at lysine 27 (H3K27me3). Moreover, inhibition of either the E3 ligase activity of polycomb‐repressive complexes 1 (PRC1) or the methyltransferase activity of polycomb‐repressive complexes 2 (PRC2) restores *Pdcd1* expression in *Cbx4*‐transfected cells. Cumulatively, this study reveals a novel function of CBX4 in the regulation of T‐cell function and expands our understanding of the epigenetic control of *Pdcd1* expression.

## Graphical abstract

E3 SUMO‐protein ligase CBX4, a key component of polycomb‐repressive complexes 1, has been reported to regulate a variety of genes implicated in tumor growth, metastasis, and angiogenesis. We found a novel function of CBX4 in the regulation of histone modification, H2AK119ub1, at the *Pdcd1* locus in activated T cells, affecting antitumor immunity. This finding helps expand our understanding of the epigenetic control of *Pdcd1* expression.

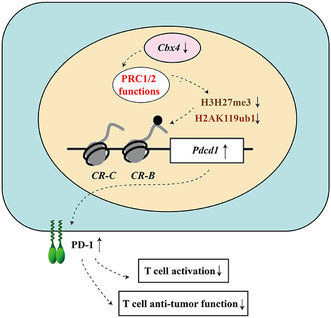

**Abbreviations**
AP‐1: activator protein‐1
BrdU: 5‐bromo‐2‐deoxyuridine
CBX4: E3 SUMO‐protein ligase
CCNE1: cyclin E1
ChIP: chromatin IP
*CR‐B*
: conserved region *‐B*
*CR‐C*
: conserved region *‐C*
CTLA‐4: cytotoxic T lymphocyte‐associated antigen‐4
EED: embryonic ectoderm development
EZH2: enhancer of Zeste homolog
FoxO1: forkhead box O1
GCN5: lysine Acetyltransferase 2A
GzmB: granzyme B
H2AK119ub1: mono‐ubiquitinated histone H2A at lysine 119
H3K27me3: trimethylated histone H3 at lysine 27
HDAC2: histone deacetylase 2
HDAC3: histone deacetylase 3
IFN‐γ: interferon gamma
LAG‐3: lymphocyte‐activation gene 3
NFATc1: nuclear factor of activated T cells 1
PBS: phosphate‐buffered saline
PD‐1: programmed cell death protein 1
PRC1: polycomb‐repressive complexes 1
PRC2: polycomb‐repressive complexes 2
SIM: SUMO‐interacting motifs
SUZ12: suppressor of Zeste 12 Homolog
TCR: T‐cell receptor
TIM‐3: T‐cell immunoglobulin domain and mucin domain‐3
TNF‐ɑ: tumor necrosis factor‐alpha
ZAP‐70: tyrosine‐protein kinase ZAP‐70

## 1 Introduction

Polycomb group proteins are crucial transcriptional repressors that mediate epigenetic gene silencing [1, 2]. They are organized into two forms of polycomb‐repressive complexes (PRC), PRC1, and PRC2. While PRC1 acts to monoubiquinate lysine 119 of histone 2A (H2AK119ub1), PRC2 mainly targets histone H3 lysine 27 for mono‐, di‐, and trimethylation. Despite the functional distinction, these two complexes are tightly interconnected and show remarkable synergism in transcriptional repression [3, 4, 5, 6]. The polycomb chromobox (CBX) family, including CBX2, CBX4, CBX6, CBX7, and CBX8, are important components of the PRC1 complex [7]. Much effort has been devoted to dissecting their role in tumorigenesis. In a context‐dependent manner, they act as either oncogenes or tumor suppressors. CBX7, for example, shows an antitumor activity in lung cancer through interacting with histone deacetylase 2 (HDAC2) and inhibiting CCNE1 expression [8]. In gastric cancer and lymphoma, however, it primarily exerts a tumor‐promoting activity, most likely by repressing the transcription of the INK4a/ARF locus [9, 10, 11]. Similarly, CBX4 plays opposite roles in colon cancer versus osteosarcoma. While inhibiting the metastasis of colon cancer by recruiting histone deacetylase 3 (HDAC3) to the *Runx2* promoter [12], CBX4 promotes the metastasis of osteosarcoma by recruiting GCN5, which catalyzes H3K27 acetylation of the *Runx2* promoter [13]. Of the CBX family members, CBX4 is of particular interest in that, in addition to the canonical polycomb function, it possesses two SUMO‐interacting motifs (SIM) and has the capacity to catalyze the sumoylation of a variety of substrates, such as CtBP [14], Dnmt3a [15], and BMI‐1 [16]. This SUMO E3 ligase activity has been demonstrated to be critical for DNA damage response [16] and epidermal stem cell activation and differentiation [17].

Adaptive immune responses, mainly mediated by T cells, have been shown to play a vital role in the control of tumor development and progression [18, 19, 20, 21]. Tumor antigens arising from somatic mutation or aberrant expression are captured and processed by antigen‐presenting cells. Peptides derived from these antigens are then displayed bound to class I or II MHC molecules for recognition by CD8^+^ and CD4^+^ T cells, respectively. As the principal component of immune protection against tumors, activated CD8^+^ T cells directly lysed tumor cells. CD4^+^ T cells, on the contrary, secrete an array of cytokines, which promote the generation and effector functions of CD8^+^ T cells. Notably, immune responses frequently fail to control tumor growth as tumors develop the capacity to evade antitumor immunity [21, 22, 23]. One of the best‐studied mechanisms is the inhibition mediated by programmed cell death protein 1 (PD‐1) on TCR signals. Physiologically, after stimulation, phosphorylation of Lck and ZAP‐70 will initiate the TCR signaling cascade. PD‐1 signals directly targeting on the phosphorylation of Lck and ZAP‐70 are normally involved in the fine‐tuning of T‐cell functions and prevention of their over‐activation [24, 25, 26, 27]. This pathway, however, is hijacked by tumors. High levels of PD‐1 expression are often detected in tumor‐infiltrating T cells and these tumor‐associated PD‐1^+^ T cells are functionally impaired [28]. Blocking this inhibitory pathway, on the contrary, leads to the development of strong T‐cell responses against a variety of tumors [29, 30, 31, 32, 33].

Previous studies of CBX proteins in tumorigenesis have been focused on their role in the regulation of malignant behaviors of tumor cells. Their impact on antitumor immunity is largely ignored. Li et al. [34] have reported that CBX7 promotes activation‐induced cell death of CD4^+^ T cells by regulating DNA demethylation of the FasL promoter. In addition, data from our group indicate that CBX4 are absolutely required for thymic organogenesis. Loss of *Cbx4* impairs the formation and maintenance of the thymic epithelium, leading to the disruption of T‐lymphopoiesis [35]. In view of the vital role of T‐cell‐mediated immunity in the protection against tumors, here we explored the regulatory role of CBX4 in T‐cell function and antitumor immunity. In a murine model of colorectal carcinoma, T‐cell‐specific deficiency of *Cbx4* resulted in accelerated tumor growth. T cells from the knockout mice were defective in the response to TCR stimulation, most likely due to dysregulated *Pdcd1* expression. We further showed that, by promoting the accumulation of H2AK119ub1 and H3K27me3 histone modifications around the *Pdcd1* promoter, CBX4 served as an epigenetic repressor for *Pdcd1* expression.

## 2 Materials and methods

### 2.1 Experimental animals

*Cbx4* ^flox/flox^ mice were gifted by Guoliang Xu at the Shanghai Institutes of Biological Sciences. CD4‐Cre mice were purchased from Jackson Lab. Mice are kept in the specific‐pathogen‐free animal facility of the Peking University Health Science Center. To generate mice with selective *Cbx4* deficiency in T cells, *Cbx4* ^flox/flox^ mice were crossed with CD4‐Cre mice, both on C57BL/6 background. *Cbx4* ^flox/flox^CD4‐Cre (called ‘knockout’ here) and the *Cbx4* ^flox/flox^ (called ‘wild‐type’ here) littermates were used at the age of 10–12 weeks (divided by sex), both sexes were used in the experiments. The experimental procedures on use and care of animals were approved by the Ethics Committee of Peking University Health Science Center.

### 2.2 Cell lines

The cell lines MC38 (RRID: CVCL_0A68), EL‐4 (RRID: CVCL_0255), and Jurkat (RRID: CVCL_0065) were used in this study and have been authenticated within the past 3 years. The cell lines of EL‐4 (RRID: CVCL_0255) and Jurkat (RRID: CVCL_0065) were purchased from ATCC (Rockefeller, MD, USA; https://www.atcc.org). The cell line of MC38 (RRID: CVCL_0A68) was purchased from BTCC (http://www.btcccell.com/). Authentication was conducted using the widely accepted method of short tandem repeat (STR) profiling to ensure the integrity and reliability of the cell lines. We confirm that all experiments conducted in this study were performed using mycoplasma‐free cells. To verify the absence of mycoplasma contamination, regular screenings were performed using polymerase chain reaction (PCR) tests specifically designed for detecting the presence of mycoplasma DNA. These screenings were conducted by a reputable laboratory with expertise in mycoplasma detection. Cells were cultured in RPMI medium 1640 (Thermo Fisher Scientific, Waltham, MA, USA) supplemented with 10% fetal calf serum at 37 °C, 5% CO_2_.

### 2.3 Reagents and antibodies

Purified anti‐CD3 and anti‐CD28 antibodies, and fluorochrome‐conjugated antibodies against PD‐1, LAG‐3, CTLA‐4, and TIM‐3 were purchased from BioLegend (San Diego, CA, USA); fluorochrome‐conjugated antibodies against CD3, CD4, CD8, CD19, TCR‐β, CD44, CD62L, CD69, CD25, and Ki‐67, and Annexin V‐7AAD apoptosis staining kit were purchased from BD Corporation (Franklin, NJ, USA); fluorochrome‐conjugated antibodies against Foxp3, IL‐2, IL‐4, IL‐17, GzmB, IFN‐γ, TNF‐α, CD45, and human PD‐1 were purchased from eBioscience (San Diego, CA, USA). The anti‐CBX4 antibody and anti‐β‐actin antibody were purchased from Proteintech (Wuhan, China). Antibodies against EED, SUZ12, and EZH2 were purchased from Cell Signaling Technology (Danvers, MA, USA). IL‐2, IL‐4, IL‐6, IL‐12, and TGF‐β1 were purchased from R&D Systems (Minneapolis, MN, USA). PRT4165 and GSK126 were purchased from Selleck (Houston, TX, USA). Human CBX4 (NM_003655) cDNA plasmid, and mouse CBX4 (NM_007625) cDNA plasmid were purchased from YouBio (Changsha, China).

### 2.4 Flow cytometry

Single‐cell suspension from the spleen, lymph nodes, or tumor tissues was incubated with the appropriate antibodies for 30 min, washed, and resuspended with PBS. Flow cytometry analysis was performed on a FACS Canto Flow Cytometer (BD Corporation) and analyzed with kaluza software (Beckman Coulter, Brea, CA, USA).

### 2.5 T‐cell sorting and culture

CD3^+^ T cells were purified from lymph nodes using CD3ε MicroBead Kit (Miltenyi Biotec, Cologne, Germany) according to the manufacturer's instructions. To isolate naïve CD4^+^ or CD8^+^ cell, single‐cell suspension from lymph nodes was stained with fluorochrome‐conjugated antibodies for 30 min at 4 °C, washed twice, and resuspended at a density of 1 × 10^8^ cells·mL^−1^. CD62L^+^CD44^low^cells (naive T cells), CD62L^−^CD44^high^ (effector memory T cells), and CD62L^+^CD44^high^ (central memory T cells) were sorted using BD FACS Aria II. T cells were then placed onto anti‐CD3‐coated 96‐well flat‐bottom plates at a density of 1 × 10^5^ cells/well with the addition of 1 μg·mL^−1^ anti‐CD28. In polarization cultures, cytokines and cytokine antibodies were added as follows: Th1 (10 ng·mL^−1^ IL‐12 and 2 ng·mL^−1^ IL‐2); Th2 (20 ng·mL^−1^ IL‐4, 2 ng·mL^−1^ IL‐2 and 10 μg·mL^−1^ anti‐IFN‐γ); Th17 (10 ng·mL^−1^ TGF‐β1, 10 μg·mL^−1^ IL‐6, 10 μg·mL^−1^ anti‐IFN‐γ, and 10 μg·mL^−1^ anti‐IL‐4); and Treg (1 ng·mL^−1^ TGF‐β1 and 10 ng·mL^−1^ IL‐2).

### 2.6 CD45RB^hi^ adoptive transfer colitis model

Half a million FACS‐purified CD4^+^CD45RB^hi^ naïve T cells were intravenously injected into *Rag1* ^−/−^ mice. The receiving mice were monitored and weighed each week. At Week 7, the mice were sacrificed and measured for colon length. Colon tissues were then fixed in Bouin's fixative solutions. Hematoxylin and eosin staining was performed on paraffin sections of the colon.

### 2.7 Tumor model

MC38 cells were cultured in the DMEM medium (Gibco, Grand Island, NY, USA) and harvested in the logarithmic phase. One million cells were subcutaneously injected into the flanks of the mice. Tumor growth was measured daily or every other day over a period of 20 days using a caliper, and the tumor volume was calculated using the following formula: Tumor volume = length × width^2^/2. For the anti‐PD‐1 therapy, tumor‐bearing mice were inoculated intraperitoneally with anti‐PD‐1 (RMP1‐14, 200 μg per mouse, dissolved in PBS) or PBS on Days 8, 11, and 14. CD3^+^ T cells were purified from draining lymph nodes using CD3ε MicroBead Kit (Miltenyi Biotec) according to the manufacturer's instructions.

### 2.8 BrdU incorporation

Cells were incubated in medium containing 0.1 mm 5‐bromo‐2‐deoxyuridine (BrdU) for 1 h. BrdU incorporation was detected by flow cytometry following staining with BrdU staining kit (eBioscience) according to manufacturers' instructions.

### 2.9 Quantitative RT‐PCR

Total RNA was isolated using TRIzol (Invitrogen, Carlsbad, CA, USA) and chloroform, and cDNA was generated using the Rever TraAce™ qPCR RT Master Mix (Toyobo, Osaka, Japan) according to the manufacturer's instructions. Quantification of mRNA was conducted by qPCR using KOD SYBR® qPCR Mix (Toyobo) with primers listed in Table S1. Reactions were performed on the Eco TM Real‐time PCR system. Results were normalized to the expression of the housekeeping gene β‐actin.

### 2.10 Chromatin immunoprecipitation for histone modification analysis

Chromatin immunoprecipitation assay was conducted with 1 × 10^7^ cells using Chromatin IP (ChIP) Assay Kit (Merck KGaA, Darmstadt, Germany) according to the manufacturer's instructions. In brief, T cells were fixed in 1% formaldehyde. Chromatin was sonicated to produce fragments of 200–1000 bp long. After preclearing by incubation with Protein A Agarose/Salmon Sperm DNA (Merck KGaA), fragmented chromatin was incubated with antibody‐bound beads. Antibodies used were anti‐H3K27me3, anti‐H2AK119ub1, or the isotype control rabbit IgG (Biodragon, Suzhou, China). After washing, DNA was eluted and cross‐linking was reversed before DNA was purified using the PCR purification kit according to the manufacturer's instructions (Tiangen, Beijing, China). Eluted DNA was quantified using qPCR. Primer sequences for ChIP‐qPCR are listed in Table S2. Fold enrichment over background was calculated using values normalized to input.

### 2.11 Western blot analysis

T cells were lysed in a cell lysis buffer containing a cocktail of protease inhibitors. Proteins were separated on SDS/PAGE gel and transferred onto a PVDF membrane. Subsequently, the PVDF membranes were probed by incubation with primary antibodies, followed by incubation with horseradish peroxidase (HRP)‐conjugated secondary antibodies. The protein bands were visualized with a SuperSignal WestPico Kit (Thermo Fisher) according to the manufacturer's instructions. Antibodies against EED (85322S; clone: E4L6E; antibody ratio 1 : 1000), SUZ12 (3737S; clone: D39F6; antibody ratio 1 : 1000), EZH2 (5246S; D2C9; antibody ratio 1 : 1000), anti‐H3K27me3 (9733S; clone: C36B11; antibody ratio 1 : 5000), and anti‐H2AK119ub1 (8240S; clone: D27C4; antibody ratio 1 : 5000) were purchased from Cell Signaling Technology.

### 2.12 Statistical analysis

Statistical analyses were performed with graphpad prism 5. Data are presented as the mean ± standard deviation (SEM). Two‐tailed Student's *t*‐test or paired *t*‐test were used for single comparison. In all tests, *P* < 0.05 were considered statistically significant.

### 2.13 Ethics statement

This study was carried out in accordance with the recommendations of and the approval (protocol No. LA2018106) by the Ethics Committee of Peking University Health Science Center.

## 3 Results

### 3.1 T‐cell‐specific deletion of *Cbx4* impairs antitumor immunity

**Fig. 1:**
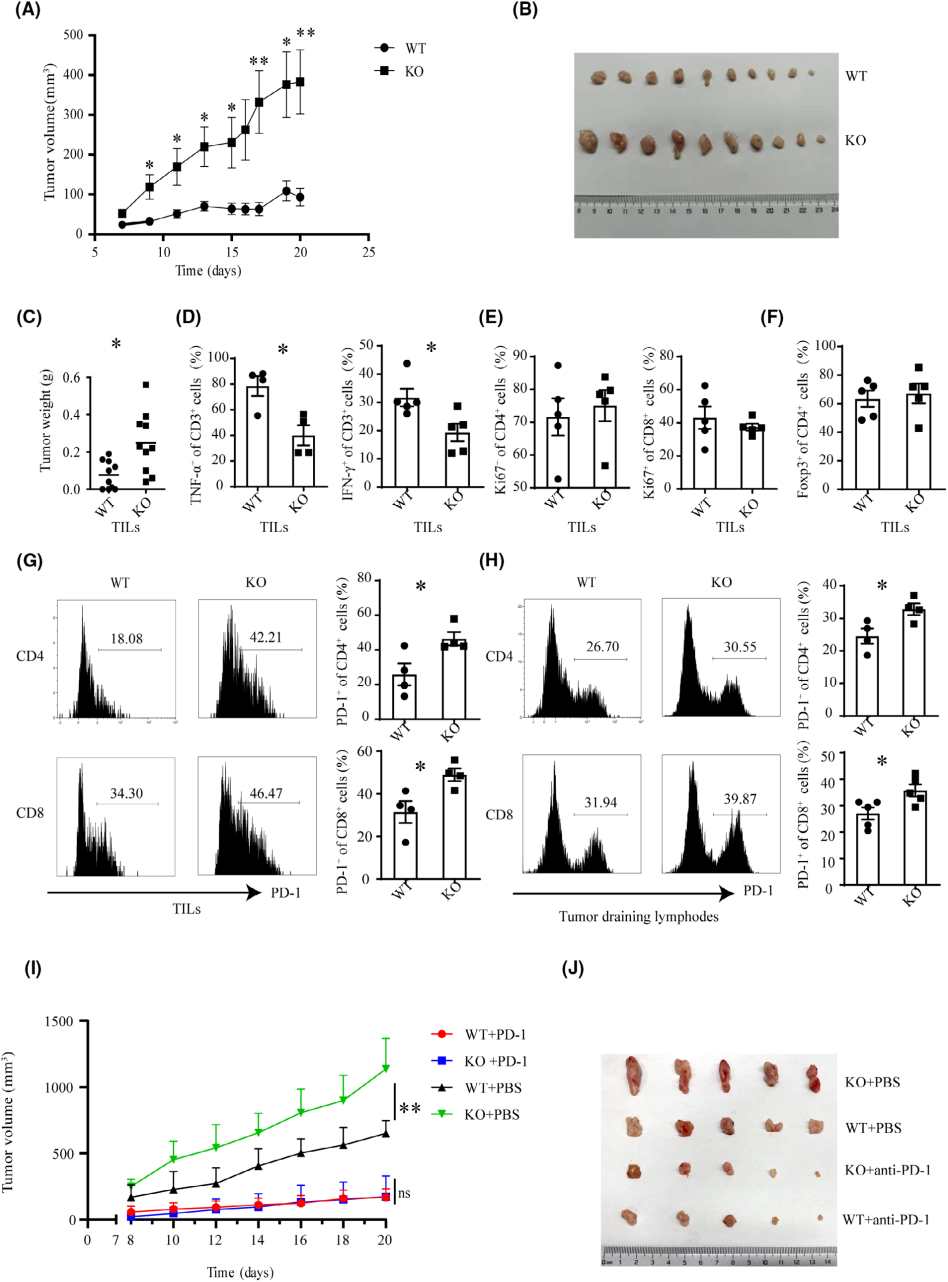
Suppressed antitumor immunity following T‐cell‐specific deletion of *Cbx4*. WT (wild‐type) and *Cbx4* KO (knockout) mice were subcutaneously injected with MC38 cells (1 × 10^6^ cells per mouse). Tumor volume was monitored over a period of 20 days. Tumors were removed at the end of experiment and weighted. Tumor‐infiltrating lymphocytes were isolated and analyzed for the expression of surface markers, intracellular cytokines, Foxp3, and Ki‐67 using flow cytometry. (A) Tumor volume over the course (*n* = 10). (B) Tumor size (scale bar = 1 cm, *n* = 10). (C) Tumor weight (*n* = 10). (D) The percentage of TNF‐α^+^ and IFN‐γ^+^ cells in CD3^+^ T cells (*n* = 4). (E) The percentage of Ki‐67^+^ cells among CD4^+^ or CD8^+^ T cells (*n* = 5). (F) The percentage of Foxp3^+^ cells in CD4^+^ T cells (*n* = 5). (G) The percentage of PD‐1^+^ cells among CD4^+^ or CD8^+^ T cells (*n* = 4). The horizontal axis represents fluorescence intensity. (H) Tumor‐draining lymph nodes were removed. Flow cytometry was performed to examine PD‐1 expression in CD4^+^ or CD8^+^ T cells (*n* = 4). The horizontal axis represents fluorescence intensity. Data are presented as Mean ± SE. Unpaired *t*‐test was used for comparison. *P < 0.05, **P < 0.01. (I and J) WT and CBX4 KO mice were subcutaneously injected with MC38 cells. Tumor growth was monitored over a period of 20 days. Mice were intraperitoneally injected with PBS or anti–PD‐1 (RMP1‐14, 200 μg per mouse, dissolved in PBS) every 3 days (three times in total) 8 days after MC38 inoculation and tumor sizes were recorded every day afterward (scale bar = 1 cm, *n* = 5), **P < 0.01, two‐way ANOVA, Statistical data are presented as Mean ± SE.

To explore the potential role of CBX4 in T‐cell‐mediated antitumor immunity, we generated T‐cell‐specific *Cbx4*‐deficient mice by crossbreeding mice with *lox*P‐flanked *Cbx4* alleles (*Cbx4* ^fl/fl^) and CD4^Cre^ transgenic mice and the efficiency and specificity of the deletion were confirmed (Fig. S1A). CD4‐specific depletion of *Cbx4* leads to its depletion not only in CD4^+^ T cells but also CD8^+^ T cells as a likely consequence of the co‐expression of CD4 and CD8 markers in DP cells, precursors of the CD4^+^ or CD8^+^ (SP) thymocytes. Flow cytometric analysis indicated that T‐cell‐specific deletion had no significant effect on thymocyte development (Fig. S1B) and the peripheral T‐cell compartment (Fig. S1C). The KO mice and the wild‐type (WT) littermates were subcutaneously inoculated with MC38 tumor cells. As measured by volume and weight, tumors in KO mice showed accelerated growth (Fig. 1A–C). At the end of observation, tumor‐infiltrating lymphocytes (TIL) were isolated and subjected to flow cytometric analysis. As shown in Fig. 1D, T cells from tumors in KO mice produced significantly lower levels of effector cytokines, such as TNF‐α and IFN‐γ (Fig. 1D). On the contrary, comparable Ki‐67 staining was observed for both CD4^+^ and CD8^+^ T cells from WT and KO mice as well as frequency of Foxp3^+^CD4^+^ cells (Fig. 1E,F), excluding the possibility of altered proliferation or Tregs recruitment. We next examined the expression of immune checkpoint inhibitors. In the absence of *Cbx4*, increased percentages of PD‐1^+^ cells were found among CD8^+^ and CD4^+^ T cells in tumor tissues (Fig. 1G) and from draining lymph nodes (Fig. 1H), whereas no difference was detected for LAG3 (Fig. S2). Collectively, these results indicate that *Cbx4* deficiency in T cells impairs antitumor immunity. To further clarify that the effect of CBX4 on T cells is through the regulation of PD‐1 in tumors, we performed anti‐PD‐1 checkpoint blockade assay to treat subcutaneous engraftment in WT and CBX4 KO mice. As indicated, PD‐1 antibody therapy resulted in significant tumor suppression both in WT and KO mice. Notably, anti‐PD‐1 therapy diminished the difference in tumor growth between WT and CBX4 KO mice (Fig. 1I,J), suggesting that the accelerated tumor development due to *Cbx4* deficiency is mainly determined by their enhanced PD‐1 expression.

### 3.2 Loss of *Cbx4* leads to upregulation of PD‐1

**Fig. 2:**
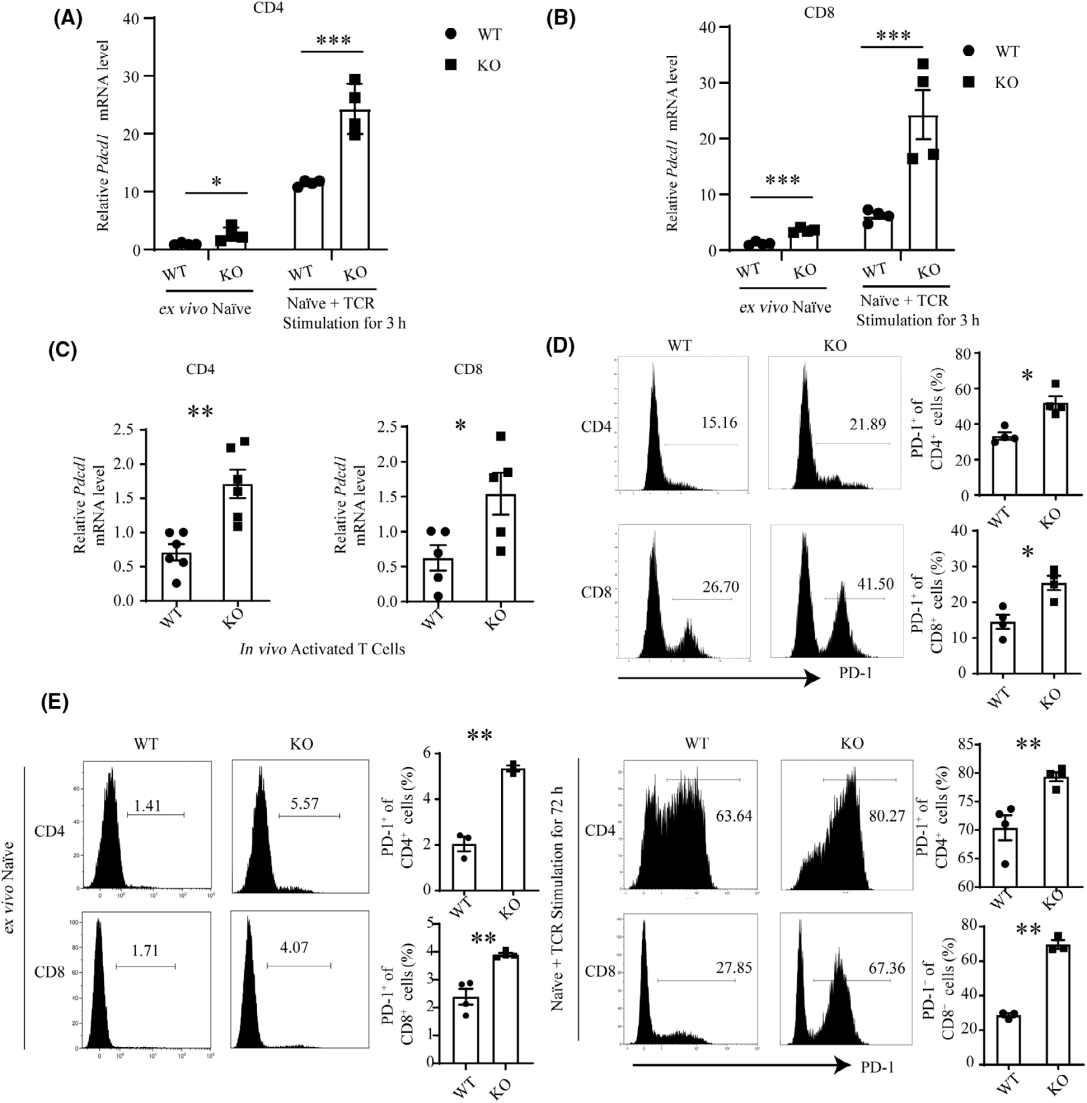
Upregulation of PD‐1 induced by *Cbx4* deficiency. (A–C) Relative *Pdcd1* mRNA levels in *Cbx4*‐deficient versus WT (wild‐type). (A) CD4^+^CD62L^+^CD44^−^ naïve or TCR‐stimulated (3 h) (*n* = 4) (B), CD8^+^CD62L^+^CD44^−^ naïve or TCR‐stimulated (3 h) (*n* = 4), (C) and freshly isolated CD44^+^ effector/memory CD4^+^ and CD8^+^ T cells as determined by RT‐qPCR (*n* = 5). (D, E) PD‐1 protein expression in *Cbx4*‐deficient versus WT splenic total (D), purified naïve, and TCR‐stimulated (72 h) (*n* = 4). (E) CD4^+^ and CD8^+^ T cells as determined by flow cytometry (*n* = 3). Representative histograms are shown on the left. Statistical data are presented as Mean ± SE on the right. Each experiment was repeated at least three times. Unpaired *t*‐test was used for comparison, *P < 0.05, **P < 0.01, ***P < 0.001.

The elevated levels of PD‐1 in tumor‐infiltrating T cells prompted us to scrutinize the regulatory effect of CBX4 on the expression of PD‐1 and other known negative regulators in resting and activated T cells. We sorted out the naïve T cells as Fig. S3A and examined whether *Cbx4* deficiency had impact on *Pdcd1* mRNA transcription. Our results showed that *Cbx4* KO naïve T cells had increased basal levels of *Pdcd1* mRNA in comparison with the WT counterparts (Fig. 2A), and the difference was maintained upon TCR stimulation (Fig. 2B). On the contrary, *Cbx4* deficiency had no significant impact on other negative regulators, including *Lag3*, *Ctla4*, *Havcr2*, *Egr2/3*, *Ikzf1*, and *Tle4* (Fig. S3B,C). Increased *Pdcd1* mRNA was also detected in purified CD44^high^CD4^+^ and CD8^+^ T cells (Fig. 2C). Flow cytometry was next engaged to analyze PD‐1 protein expression. In line with mRNA expression, PD‐1^+^ cells were present at an increased frequency among *Cbx4*‐deficient T cells (Fig. 2D), including central and effector memory cells enriched as Fig. S3A (Fig. S4A,B). When stimulated with anti‐CD3 and anti‐CD28 *in vitro*, naïve T cells quickly upregulated PD‐1 expression and significantly more PD‐1^+^ cells were generated in the cultures of *Cbx4*‐deficient T cells (Fig. 2E). Other than PD‐1 TIM‐3, LAG‐3 and CTLA‐4 are also important inhibitory roles in T‐cell function. In the process of rapid T‐cell activation, TIM‐3, LAG‐3, and CTLA‐4 will make sure T cells are not overactivated. Our data demonstrated there was comparable expression of TIM3, LAG3, and CTLA4 in WT and KO peripheral T cells (Fig. S4C,D), suggesting the effect of *Cbx4* was relatively specific for PD‐1. So was the frequency of CD25^+^Foxp3^+^ Treg cells and the PD‐1 expression in these cells (Fig. S4E).

**Fig. 3:**
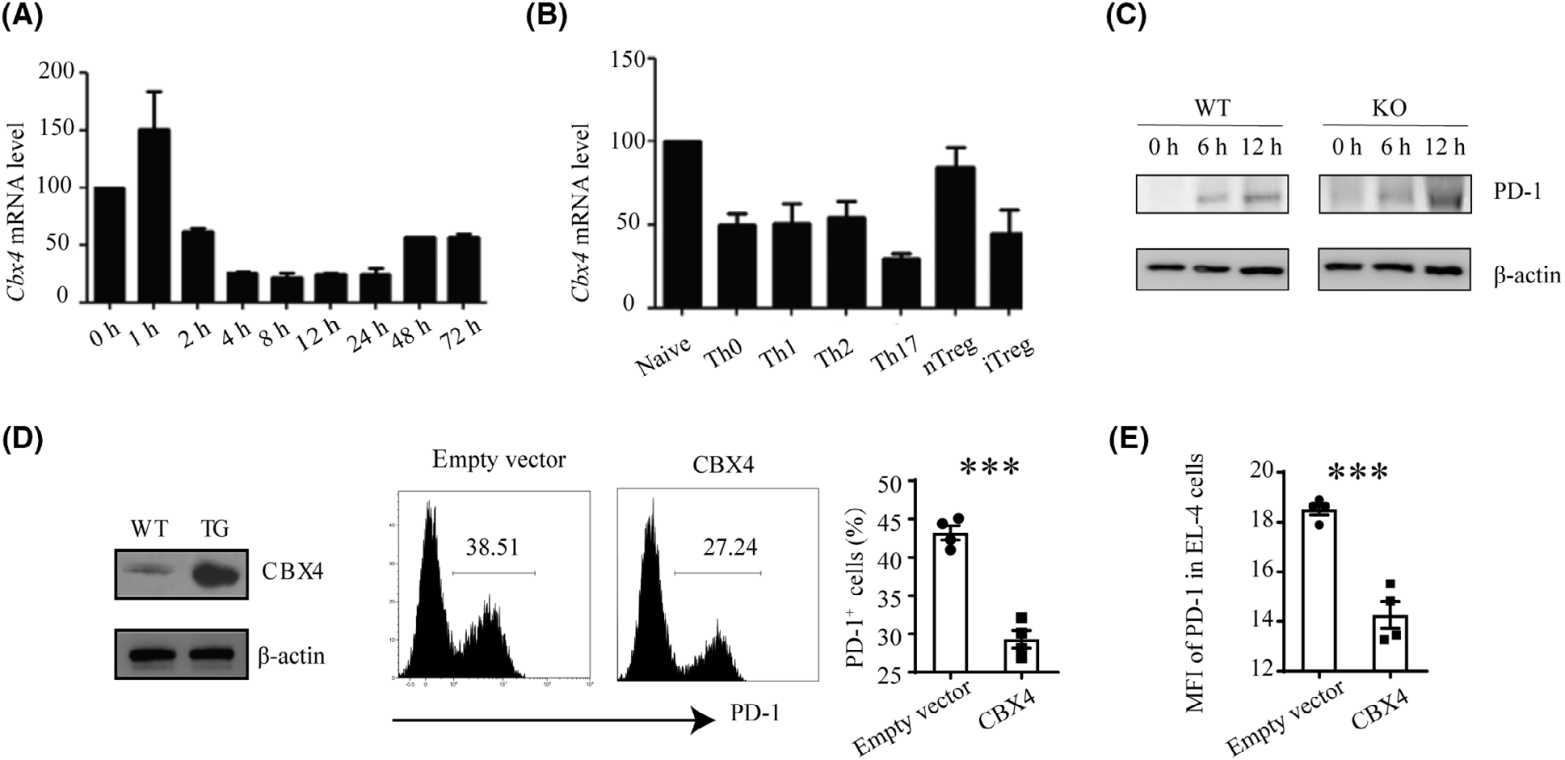
Inhibition of PD‐1 expression by CBX4. (A) Relative levels of *Cbx4* mRNA expression in naïve (0 h) versus TCR‐stimulated CD4^+^ T cells at different time points as measured by quantitative RT‐PCR. Data from three independent experiments are shown. Error bars indicate SEM. (B) Relative levels of *Cbx4* mRNA expression in naïve T cells, naturally occurring Treg (nTreg) cells, and effector T cells generated under various polarizing conditions for 72 h. The experiment was repeated three times. (C) Naive CD4^+^ T cells from WT and KO mice were stimulated with anti‐CD3 (2 μg·mL^−1^) and anti‐CD28 (1 μg·mL^−1^) for 12 h. PD‐1 levels were detected 6 h and 12 h after stimulation by western blotting (*n* = 2). (D) Jurkat cells were transfected with Myc‐CBX4 or empty vectors. CBX4 expression was verified western blotting (left). Following stimulation with anti‐CD3 (2 μg·mL^−1^) and anti‐CD28 (1 μg·mL^−1^) for 24 h, PD‐1 expression in the transfectants was detected by flow cytometry (*n* = 4). (E) EL‐4 cells were transfected with Flag‐CBX4 or empty vectors. Following stimulation with anti‐CD3 (2 μg·mL^−1^) and anti‐CD28 (1 μg·mL^−1^) for 24 h, PD‐1 expression in the transfectants was detected by flow cytometry (*n* = 4). Representative histograms are shown in the middle. Statistical data are presented as Mean ± SE Each experiment was repeated at least three times. Unpaired *t*‐test was used for comparison, ***P < 0.001.

To further elucidate the regulatory role of CBX4, we analyzed its expression over the course of T‐cell activation. *Cbx4* mRNA was seen to be briefly upregulated at the early stage, however reduced below basal levels thereafter (Fig. 3A). Accordingly, all effector T‐cell subsets, including Th0, Th1, Th2, Th17, and iTreg displayed low expression of *Cbx4* (Fig. 3B). Importantly, after stimulation, PD‐1 expression upregulation was consistent with *Cbx4* expression pattern, although higher PD‐1 expression was found in KO T cells all the time points (Fig. 3C). In support of a direct role of CBX4 in PD‐1 expression, transfection of Jurkat cells and EL‐4 cells with a *Cbx4*‐expression construct were produced and purified respectively (Fig. S5A). High expression of *Cbx4* reduced the level of PD‐1 on their surface (Fig. 3D,E).

### 3.3 *Cbx4*‐deficient T cells exhibit activation defects

**Fig. 4:**
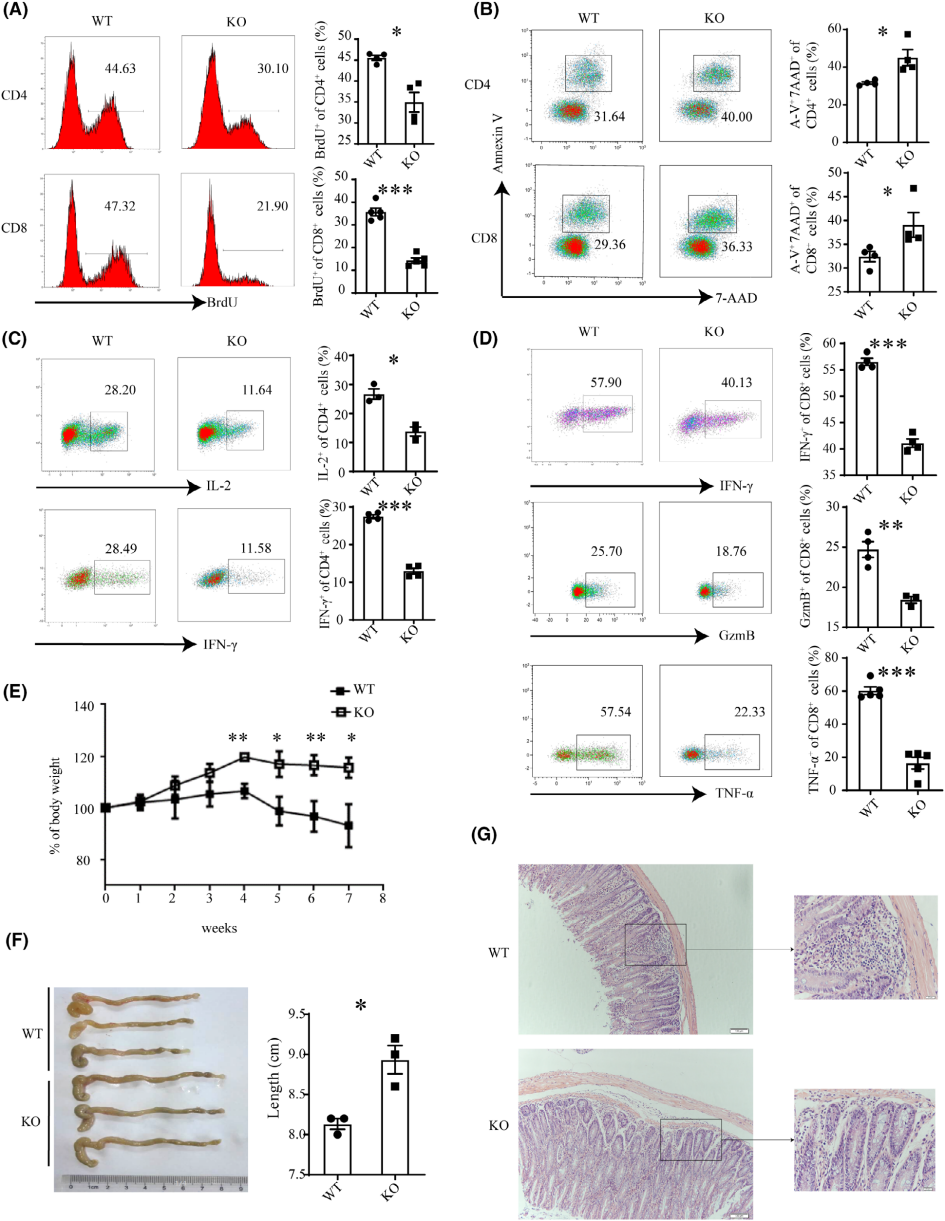
Activation defects of *Cbx4*‐deficient T cells. Naive CD4^+^ and CD8^+^ T cells from WT (wild‐type) and KO (knockout) mice were stimulated with anti‐CD3 (2 μg·mL^−1^) and anti‐CD28 (1 μg·mL^−1^) for 72 h. (A–D) Cell proliferation was measured by BrdU incorporation (A). Cell survival was analyzed with Annexin V and 7‐AAD staining (*n* = 4) (B). IL‐2 and IFN‐γ production by CD4^+^ T cells (*n* = 4) (C) and IFN‐γ, GzmB, and TNF‐α expression by CD8^+^ T cells (*n* = 4) (D) was examined by flow cytometry. Representative histograms (left) and statistical data (right) from three to four independent experiments are shown. (E–G) Body weight of *Rag1* ^−/−^recipient mice given wild‐type or *Cbx4* KO CD4^+^CD45RB^hi^ naïve T cells were adoptively transferred into *Rag1* ^−/−^recipient mice (*n* = 3 for each group). Body weight was monitored weekly (E). At the end of experiment, colon length was recorded (F) and the colonic sections were examined by HE staining (scale bar = 1 cm) (G). Scale bar = 100 μm. Data are presented as Mean ± SE. Unpaired *t*‐test was used for comparison, *P < 0.05, **P < 0.01, ***P < 0.001.

We next investigated the impact of dysregulated PD‐1 expression on T‐cell activation. Naïve T cells were purified and stimulated with anti‐CD3 and anti‐CD28. In comparison with WT cells, both CD4^+^ and CD8^+^ T cells from KO mice showed reduced proliferation as measured by BrdU incorporation (Fig. 4A) and slightly increased apoptosis in Annexin V/7‐AAD staining (Fig. 4B). Moreover, *Cbx4* KO CD4^+^ T cells produced less Th1 cytokines, such as IL‐2 and IFN‐γ (Fig. 4C). On the contrary, differentiation into Th2, Treg, or Th17 cells was not affected (Fig. S5B). Impaired function was also observed for *Cbx4* KO CD8^+^ T cells in terms of IFN‐γ, GzmB, and TNF‐α expression (Fig. 4D). To reveal the functional consequence of the activation defect, a murine model of colitis was established by transferring CD4^+^CD45RB^hi^ naïve T cells into *Rag1*‐deficient hosts. Since the ratio of CD4^+^ in CD45^+^ cells showed no difference in peripheral blood in the course of colitis, we believed *Rag1‐*deficient mice received similar donor cells from WT and KO mice (Fig. S5C). Adoptive transfer of WT T cells caused rapid weight loss (Fig. 4E), significant colon shortening (Fig. 4F, Fig. S5D), and apparent more severe colonic pathomorphology, including more transmural inflammation, increased proliferation, crypt abscesses, and obvious infiltration (Fig. 4G). Minimal signs of colitis, however, were observed following adoptive transfer of KO T cells (Fig. 4E–G), indicating that they were less pathogenic. Briefly, these results support that CBX4 is required for the proper activation of naïve T cells.

### 3.4 CBX4 promotes the formation of inhibitory histone modifications at the *Pdcd1* locus

**Fig. 5:**
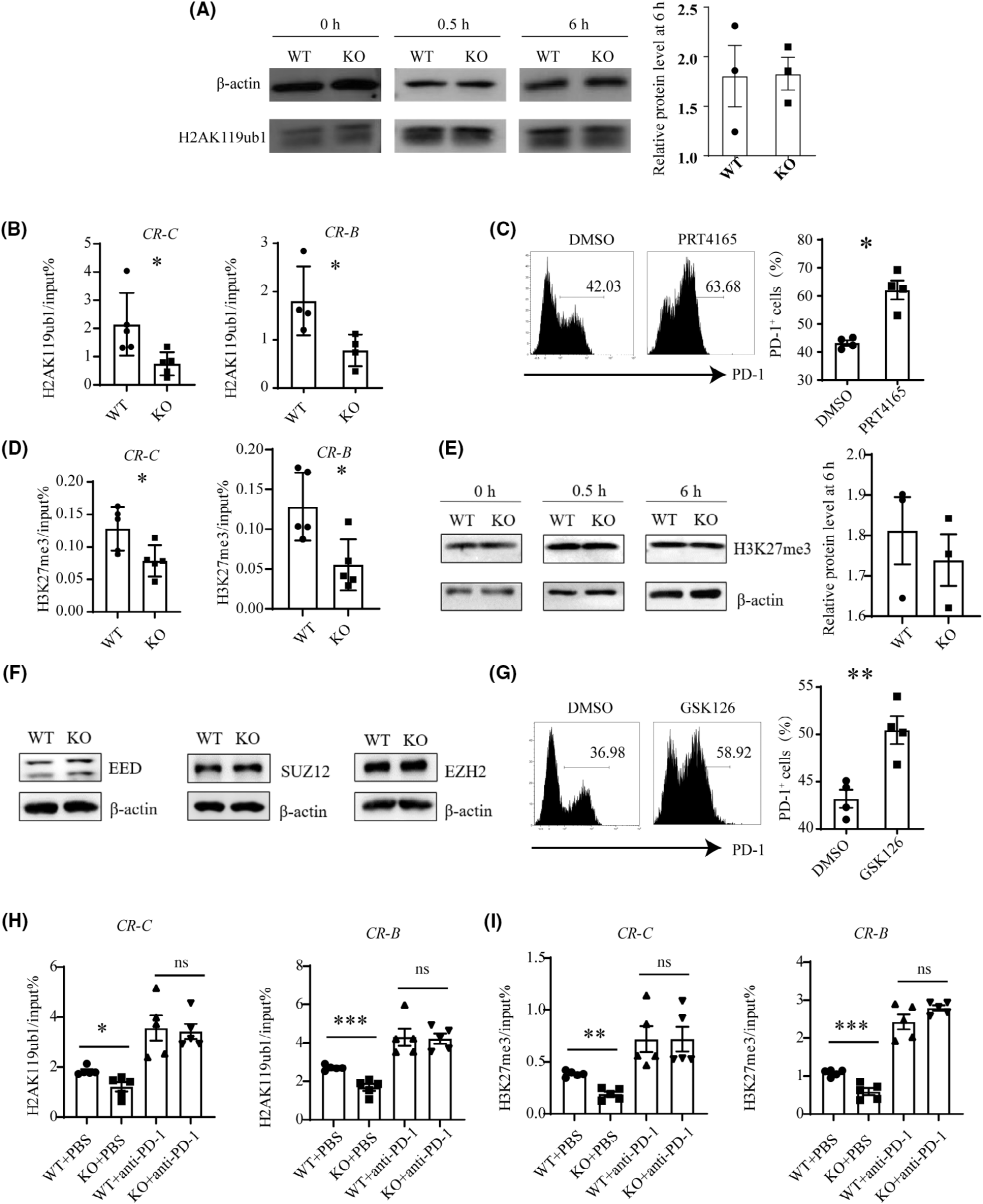
Reduced deposition of inhibitory histone modifications at the *Pdcd1* locus in *Cbx4*‐deficient activated T cells. (A and B): (A) MACS‐purified CD3^+^ T cells from WT (knockout) and *Cbx4* KO (knockout) mice were stimulated with anti‐CD3 and anti‐CD28. Total H2AK119ub1 levels were detected before and 0.5 h and 6 h after stimulation by western blotting. Representative results are shown from three independent experiments, and relative protein level was shown on the right side at the point of 6 h (*n* = 3). (B) H2AK119ub1 modification at *CR‐C* and *CR‐B* of the *Pdcd1* locus at 0.5 h were analyzed by ChIP‐qPCR. Six and five pairs of WT and KO mice were used for the analysis of the *CR‐C* and *CR‐B* site, respectively (*n* = 4). (C) PD‐1 expression on Jurkat cells stimulated with anti‐CD3 and CD‐28 for 24 h with the addition of PRT4165 or DMSO (*n* = 5). (D) Levels of H3K27me3 modification at the *CR‐C* and *CR‐B* site (*n* = 5). (E) Total H3K27me3 levels in activated T cells were shown on the left, and relative protein level at 6 h was shown on the right side. Representative blots of three independent experiments are shown. (F) Expression of PRC2 components in T cells treated with anti‐CD3 and CD28 for 0.5 h. Representative blots of three independent experiments are shown. (G) PD‐1 expression on TCR‐stimulated Jurkat cells in the presence or absence of GSK126 (*n* = 4). (H) Levels of H2AK119ub1 modification at the *CR‐C* and *CR‐B* site in CD3^+^ T cells of draining lymph nodes in MC38 subcutaneous tumor models (*n* = 5). (I) Levels of H3K27me3 modification at the *CR‐C* and *CR‐B* site in CD3^+^ T cells of draining lymph nodes in MC38 subcutaneous tumor models (*n* = 5). Statistical data are presented as Mean ± SE. Each experiment was repeated at least three times. Unpaired *t*‐test for A–E, G–I. ns, not significant, *P < 0.05, **P < 0.01, ***P < 0.001.

CBX4 primarily regulates gene transcription through epigenetic mechanisms. Therefore, our subsequent studies were focused on altered epigenetic modifications associated with *Cbx4* deficiency. It is well established that PRC1 complexes mainly catalyzes the deposition of monoubiquitination of lysine 119 of histone H2A (H2AK119ub1). There was no significant difference in total H2AK119ub1 levels between *Cbx4* KO and WT T cells at all the time points we tested, including before or 0.5 h and 6 h after TCR engagement (Fig. 5A). ChIP analysis, however, demonstrated that H2AK119ub1 deposition was significantly reduced at the *CR‐C* and *CR‐B* sites in the *Pdcd1* promoter region in the absence of *Cbx4* (Fig. 5B) and *con*, a genomic region void of enrichment as a negative control for site‐specific enrichment of H2AK119ub1 showed no difference (Fig. S6G). PRT4165 is an inhibitor of PRC1‐mediated H2A ubiquitylation [36]. When added into cultures of activated Jurkat or EL‐4 cells, either percentage or intensity of PD‐1 expression was seen to be enhanced (Fig. 5C, Fig. S6A). Furthermore, PRT4165 overcame the suppressive effect on PD‐1 expression induced by enforced expression of *Cbx4* (Fig. S6B,C). These data support a critical role of the CBX4‐induced H2A119ub1 modification in the control of PD‐1 expression.

H2AK119 ubiquitination has been reported to facilitate the recruitment of PRC2 complex and stimulate its methyltransferase activity, leading to the trimethylation of lysine 27 of histone H3 (H3K27me3) [15, 17]. Indeed, KO T cells showed less accumulation of H3K27me3 at the *CR‐C* and *CR‐B* sites around the *Pdcd1* promoter (Fig. 5D), while they showed similar accumulation of H3K27me3 at the *con* site as a control (Fig. S6G). Again, this effect appears to be specific to *Pdcd1* as total H3K27me3 levels were comparable in WT and KO T cells, either in the resting state or upon activation (Fig. 5E). Besides, the major components of the PRC2 complex, such as EZH2, EED, and SUZ12, were equally present in WT and KO cells (Fig. 5F). To verify the functional relevance of the altered H3K27me3 modification at the *Pdcd1* promoter, GSK126 was used to block the methyltransferase activity of EZH2 [37]. Like PRT4165, GSK126 further increased PD‐1 expression on Jurkat or EL‐4 cells following TCR stimulation and counteracted the inhibitory effect on PD‐1 expression imposed by *Cbx4* overexpression (Fig. 5G, Fig. S6D–F).

To further confirm the effect of *Cbx4* on *Pdcd1* promoter region in tumor, we sorted out CD3^+^ T cells in draining lymph nodes from MC38 tumor‐bearing mice and checked their H2AK119ub1 and H3K27me3 deposition in the *Pdcd1* promoter region. As expected, in line with higher PD‐1 expression, statistically less H2AK119ub1 and H3K27me3 deposition at the *CR‐C* and *CR‐B* sites in the *Pdcd1* promoter region was found in CBX4 KO CD3^+^ T cells (Fig. 5H,I). Collectively, these data suggest that *Cbx4*‐induced downregulation of PD‐1 is largely attributable to the accumulation of inhibitory histone modifications at the promoter.

## 4 Discussion

CBX4 has been reported to have a cell‐intrinsic role in the regulation of tumor growth and invasion [12, 13, 38, 39]. From a new prospect, the present study investigated the possibility that CBX4 had additional roles in tumorigenesis by modulating T‐cell‐mediated antitumor immunity. Indeed, tumor growth was found to be accelerated in mice with T‐cell‐specific deletion of *Cbx4* and the tumor‐infiltrating T cells from these mice were defective in IFN‐γ and TNF‐α production. When stimulated with anti‐CD3 and anti‐CD28 *in vitro*, *Cbx4*‐deficient naive T cells showed impaired proliferation and reduced expression of multiple effector factors. Moreover, the knockout mice were more susceptible to lymphopenia‐induced colitis. These results highlight a novel function of CBX4 as a negative regulator of T‐cell responses.

Accumulating evidence suggests the importance of PD‐1 signaling in tumor evasion of immune attacks. Blockade of this pathway elicits durable antitumor responses and long‐term remissions in a subset of patients with a broad spectrum of cancers [40, 41, 42, 43, 44, 45, 46, 47, 48, 49]. Along with the impaired antitumor immunity, increased levels of PD‐1 were detected in tumor‐infiltrating CD4^+^ and CD8^+^ T cells from *Cbx4* knockout mice. In fact, higher percentages of PD‐1^+^ cells were observed in all major subsets of peripheral T cells or upon TCR‐mediated T‐cell activation in the absence of *Cbx4*. In contrast, enforced expression of *Cbx4* in Jurkat cells resulted in downregulation of PD‐1, rendering further support for a direct role of CBX4 in the regulation of PD‐1 expression. While the immune suppressive effect of PD‐1 is well established, its contribution to the dampened T‐cell responses in the absence of *Cbx4* remains to be verified. TCR signal transduction is an important condition for T‐cell activation, and the phosphorylation of downstream kinases Lck and ZAP‐70 of TCR is the initial signal of T‐cell cascade signaling pathway. Further investigation is needed to determine whether this effect is dependent or independent of the enhanced PD‐1 expression.

PD‐1 expression is dynamically regulated in T cells. Upon acute antigen exposure, its expression is transiently induced, but is quickly downregulated to allow for normal T‐cell responses. Chronic antigen stimulation, however, leads to sustained PD‐1 expression and immune exhaustion. A number of *cis*‐DNA elements, transcription factors, and epigenetic components are implicated in the transcriptional control of *Pdcd1* [50]. Two conserved regions (*CR‐B* and *CR‐C*), located 100‐bp and 1.1‐kb upstream of the transcription start site of *Pdcd1*, are essential for the observed patterns of gene expression [51]. Transcription factors AP‐1 [52], NFATc1 [51], and FoxO1 [53] bind to these sites to activate *Pdcd1* expression, whereas T‐bet [54] and Blimp‐1 [55] primarily function as repressors. As much as epigenetic mechanisms are concerned, an inverse correlation has been documented between PD‐1 expression and DNA methylation [56]. The landscape of histone modifications is more complicated. Briefly, H3K9ac and H3K27ac enrichment at the *CR‐C* site and the promoter region is associated with PD‐1 induction [55, 56], whereas loss of PD‐1 expression is accompanied by the appearance of repressive marks, such as H3K9me3, H3K27me3, and H4K20me3 at *CR‐C* [55]. Here, we demonstrated the presence of another form of histone modification, H2AK119ub1, at the *Pdcd1* locus in activated T cells, which was reduced in the absence of *Cbx4*. In addition, *Cbx4* deficiency was featured by less deposition of H3K27me3 at *CR‐C* and *CR‐B*. On the contrary, levels of histone acetylation, including H3ac, H4ac, H3K27ac, and H3K9ac, were not affected by *Cbx4* deficiency (data not shown), indicative of the dominant effect of repressive histone modifications. In support of the importance of CBX4‐induced histone modifications, inhibition of H2A ubiquitylation restored *Pdcd1* expression under conditions of *Cbx4* overexpression. Of note, the reduction in H2AK119ub1 and H3K27me3 appeared to be rather selective for the *Pdcd1* locus, as their total levels were comparable in WT and KO T cells. The mechanism underlying the selectivity is unclear. Chromatin immunoprecipitation assay detected no direct interaction of CBX4 with the *Pdcd1* promoter (data not shown). It is most likely that CBX4 is targeted to *Pdcd1* through other factors.

## 5 Conclusions

In conclusion, the present study reveals a previously unrecognized function of CBX4 in the regulation of T‐cell responses and antitumor immunity. The finding that CBX4‐mediated H2AK119ub1 modification regulates *Pdcd1* expression expands our understanding of the epigenetic control of antitumor immunity. This, and recent studies of the enhanced antitumor immunity with EZH2 inhibitors [57], highlights the potential of targeting epigenetic mechanisms in tumor therapy.

## Funding information

This work was supported by grants from Beijing Municipal Natural Science Foundation (7212058), the National Natural Sciences Foundation of China (31970840, 31872735 and 32230037), and Clinical Medicine Plus X‐Young Scholars Project, Peking University, the Fundamental Research Funds for the Central Universities (71006Y2823 and 71006Y3025).

## Conflict of interest

The authors declare no conflict of interest.

## Author contributions

LR, ZL, YZhou, YZhang, and WW designed the project. LR, ZL, and WW did the experiment. LR, YZhang, and WW wrote the manuscript. YJ, ZW, YZhou, and YZhao helped with mouse breeding and establishment of disease models. ZZ and JZ contributed to data analysis. XP and XS helped to perform the flow cytometric analysis and sorting.

### Peer review

The peer review history for this article is available at https://www.webofscience.com/api/gateway/wos/peer‐review/10.1002/1878‐0261.13516.

## Supporting information


**Table S1.** Primer sequences for RT‐PCR.
**Table S2.** Primer sequences for ChIP‐qPCR.
**Fig. S1.** (related to Fig. 1). Absence of *Cbx4* did not affect T‐cell development.
**Fig. S2.** (related to Fig. 1). LAG‐3 expression in tumor‐infiltrating CD4^+^ and CD8^+^ T cells from WT and *Cbx4* KO mice.
**Fig. S3.** (related to Fig. 2). *Cbx4* deficiency had no significant impact on other negative regulators.
**Fig. S4.** (related to Fig. 2). PD‐1^+^ cells were present at an increased frequency among *Cbx4*‐deficient T cells.
**Fig. S5.** (related to Figs 3, 4). *Cbx4*‐deficient T cells exhibit activation defects.
**Fig. S6.** (related to Fig. 5). CBX4 promotes the formation of inhibitory histone modifications at the *Pdcd1* locus.

## Data Availability

The data supporting the findings of this study are available from the corresponding author upon reasonable request.
