# Supplementary material for: CBX4 promotes antitumor immunity by suppressing Pdcd1 expression in T cells
Source: Mol Oncol. 2023 Oct 9;17(12):2694–708. doi: 10.1002/1878-0261.13516 (PMC10701776; doi:10.1002/1878-0261.13516)
Supplement: Supplementary file 1 — Table S1. Primer sequences for RT‐PCR. Table S2. Primer sequences for ChIP‐qPCR. Fig. S1. (related to Fig. 1). Absence of Cbx4 did not affect T‐cell development. Fig. S2. (related to Fig. 1). LAG‐3 expression in tumor‐infiltrating CD4+ and CD8+ T cells from WT and Cbx4 KO mice. Fig. S3. (related to Fig. 2). Cbx4 deficiency had no significant impact on other negative regulators. Fig. S4. (related to Fig. 2). PD‐1+ cells were present at an increased frequency among Cbx4‐deficient T cells. Fig. S5. (related to Figs 3, 4). Cbx4‐deficient T cells exhibit activation defects. Fig. S6. (related to Fig. 5). CBX4 promotes the formation of inhibitory histone modifications at the Pdcd1 locus. [file MOL2-17-2694-s001.zip › mol213516-sup-0001-AppendixS1.pdf]

Supplementary Table S1. Primer sequences for RT-PCR

**Region**	**PCR primers 5'-3'**
Actb	F:GGCTGTATTCCCCTCCATCG
	R:CCAGTTGGTAACAATGCCATGT
Pdcd1	F:CATTTGCTCCCTCTGACACTG
	R:ACCCTGGTCATTCACTTGGG
Havcr2	F:TCAGGTCTTACCCTCAACTGTG
	R:GGGCAGATAGGCATTTTTACCA
Lag3	F:CTGGGACTGCTTTGGGAAG
	R:GGTTGATGTTGCCAGATAACCC
Ctla4	F:TTTTGTAGCCCTGCTCACTCT
	R:CTGAAGGTTGGGTCACCTGTA
Egr2	F:GCCAAGGCCGTAGACAAAATC
	R:CCACTCCGTTCATCTGGTCA
Egr3	F:CCGGTGACCATGAGCAGTTT
	R:TAATGGGCTACCGAGTCGCT
Ikzf1	F:ATGTCCCAAGTTTCAGGAAAGG
	R:GCACGCCCATTCTCTTCATC
Tle4	F:CTGGACAGGTGGTTTGGACAA
	R:GAGGTGAAGTCATGTTGCTGC
Cbx4	F:AAGAAGCGGATACGCAAGGG
	R:GGAGGAGTCTTGAAGCCCAG

Supplementary Table S2. Primer sequences for ChIP-qPCR

***Pdcd1* Region**	**PCR primers 5'-3'**
CR-B	F:GGCAGTGTCGCCTTCAGTAGC
	R:CCACCTCTAGTTGCCTGTTCTC
CR-C	F:CCTCACCTCCTGCTTGTCTCTC
	R:GTGAGACCCACACATCTCATTGC
con	F:ATTCCCATCCATACCTTGCTCC
	R:ATCGAGCTGTGCTGATGGACAC

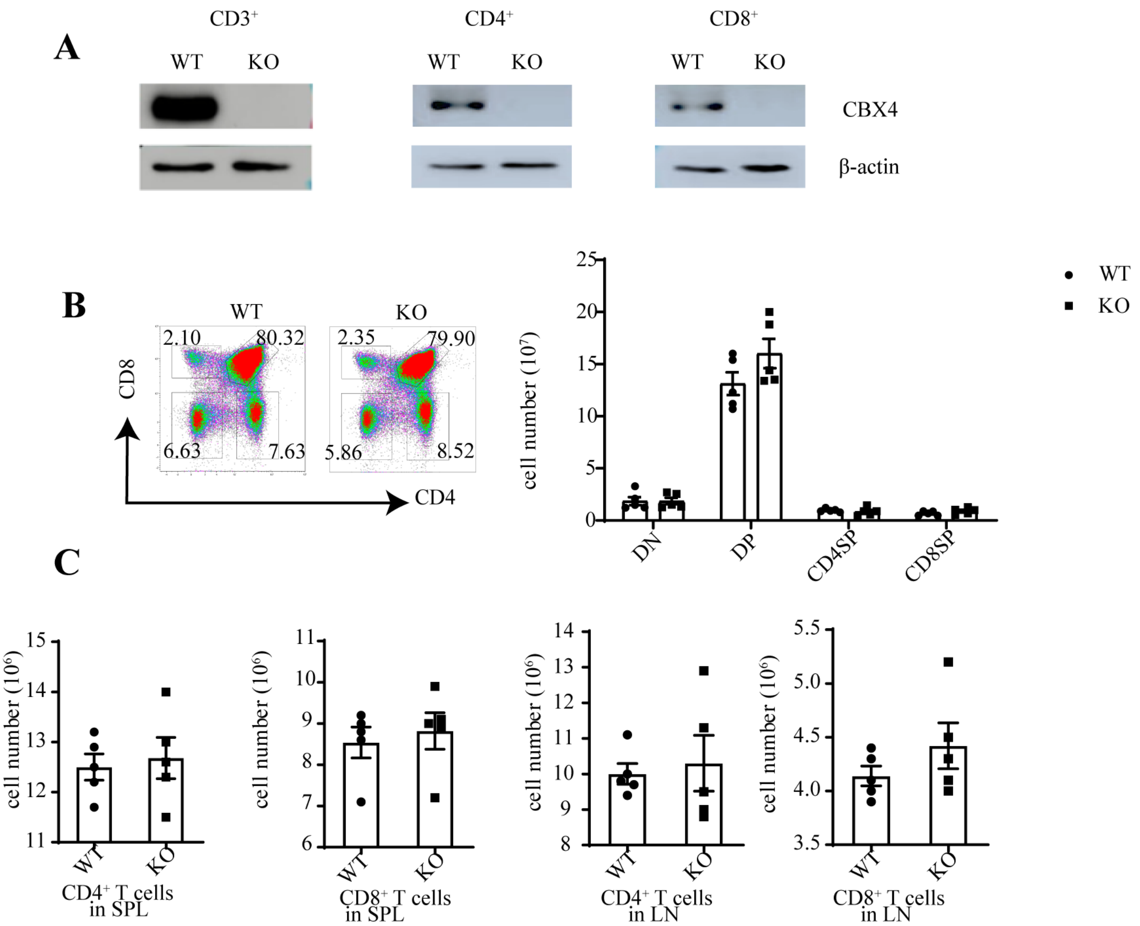

Supplementary Figure S1

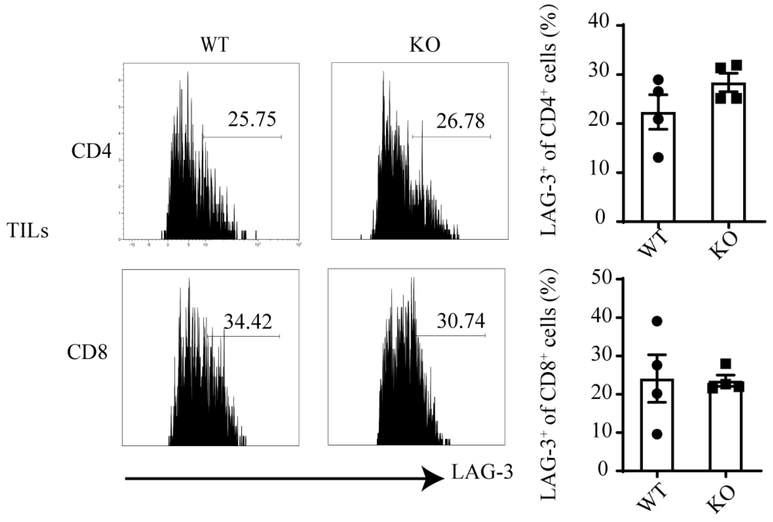

Supplementary Figure S2

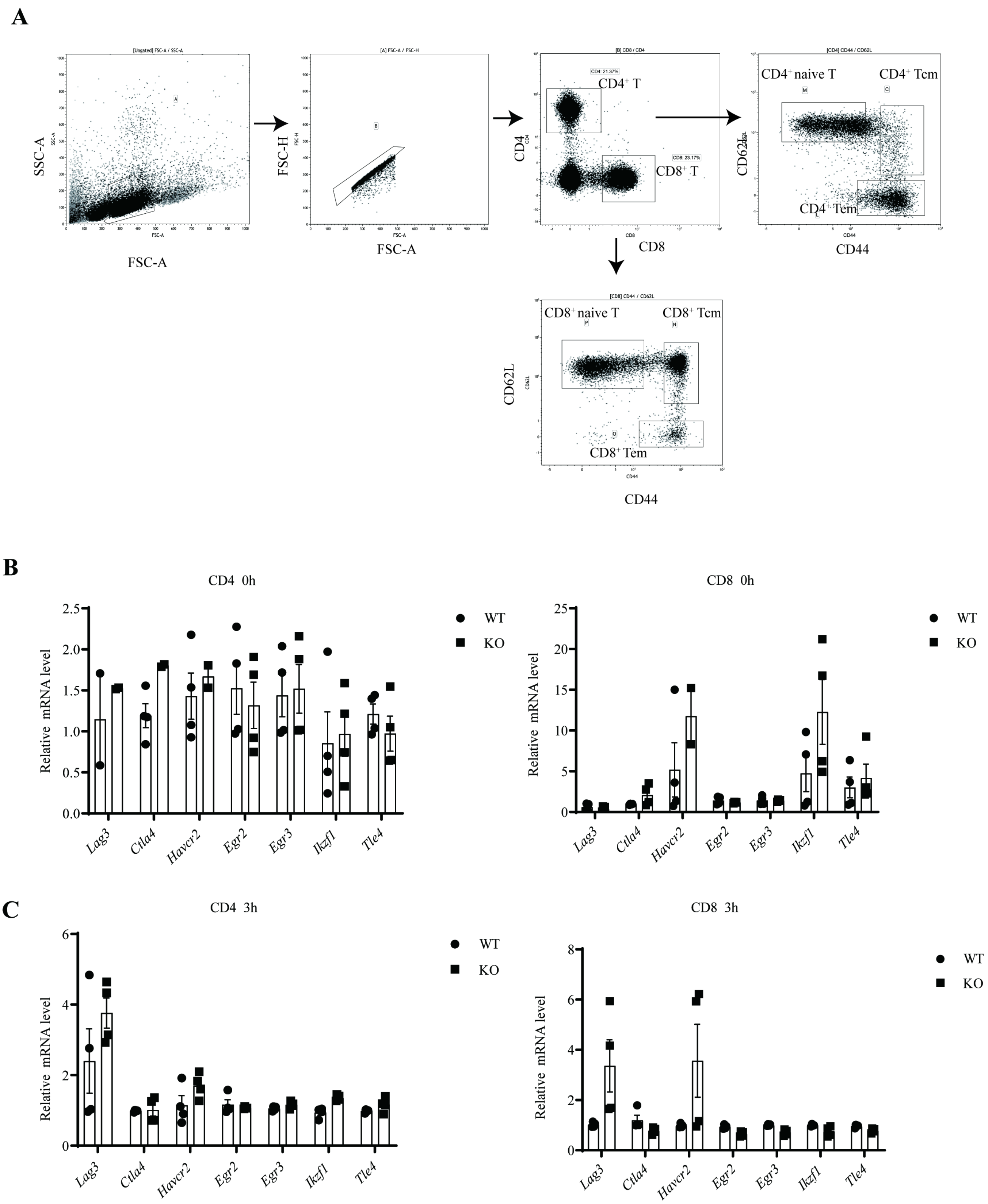

Supplementary Figure S3

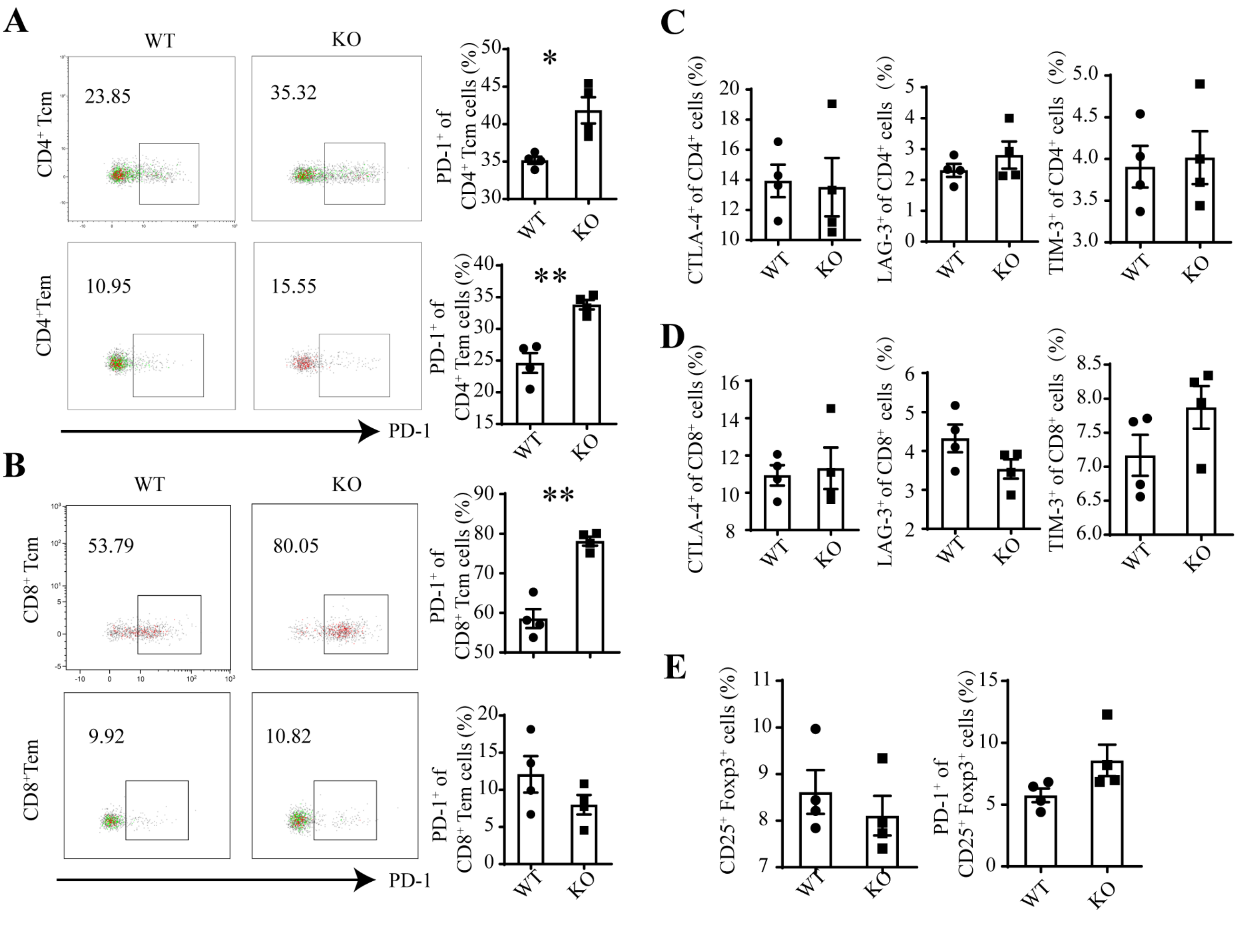

Supplementary Figure S4

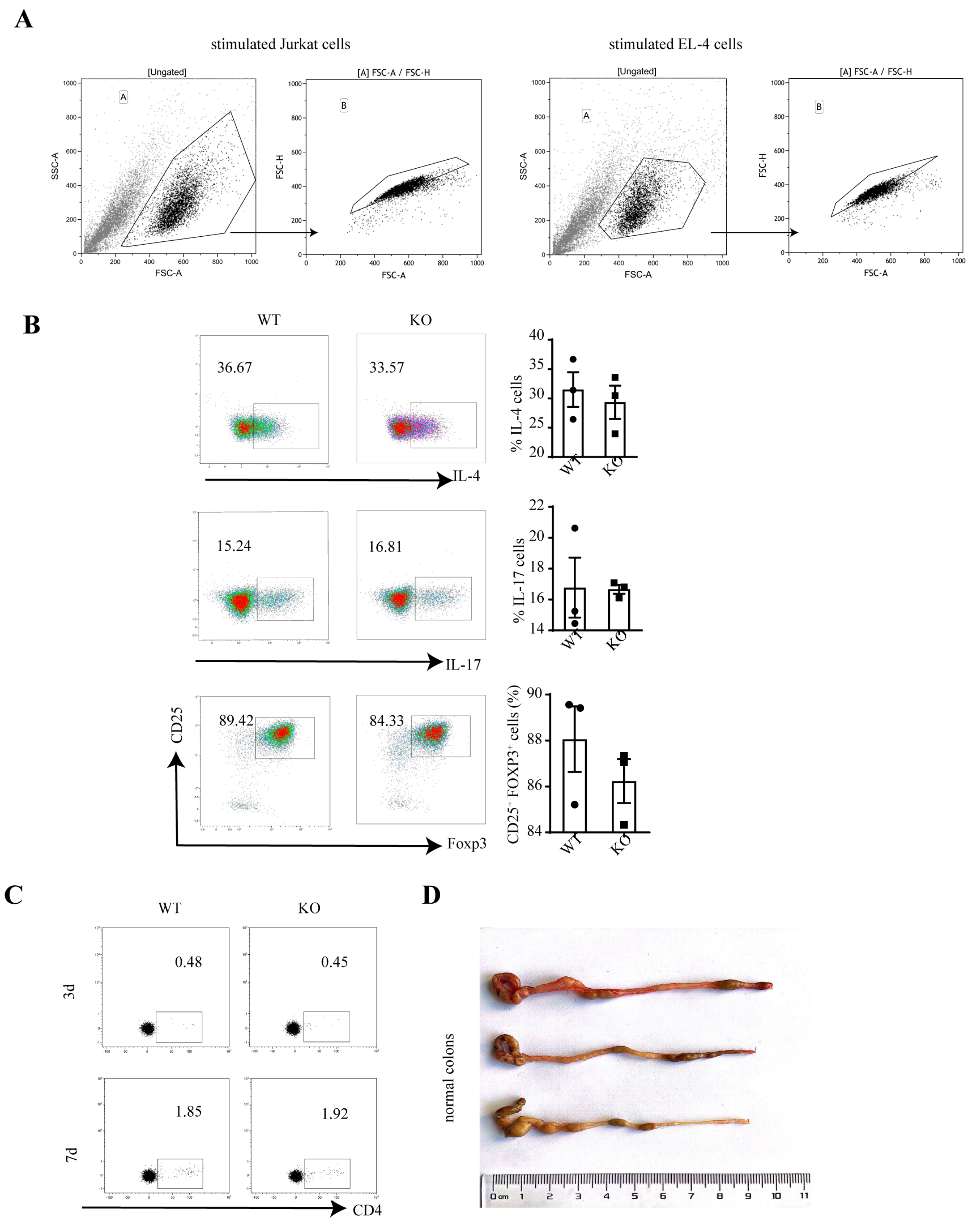

Supplementary Figure S5

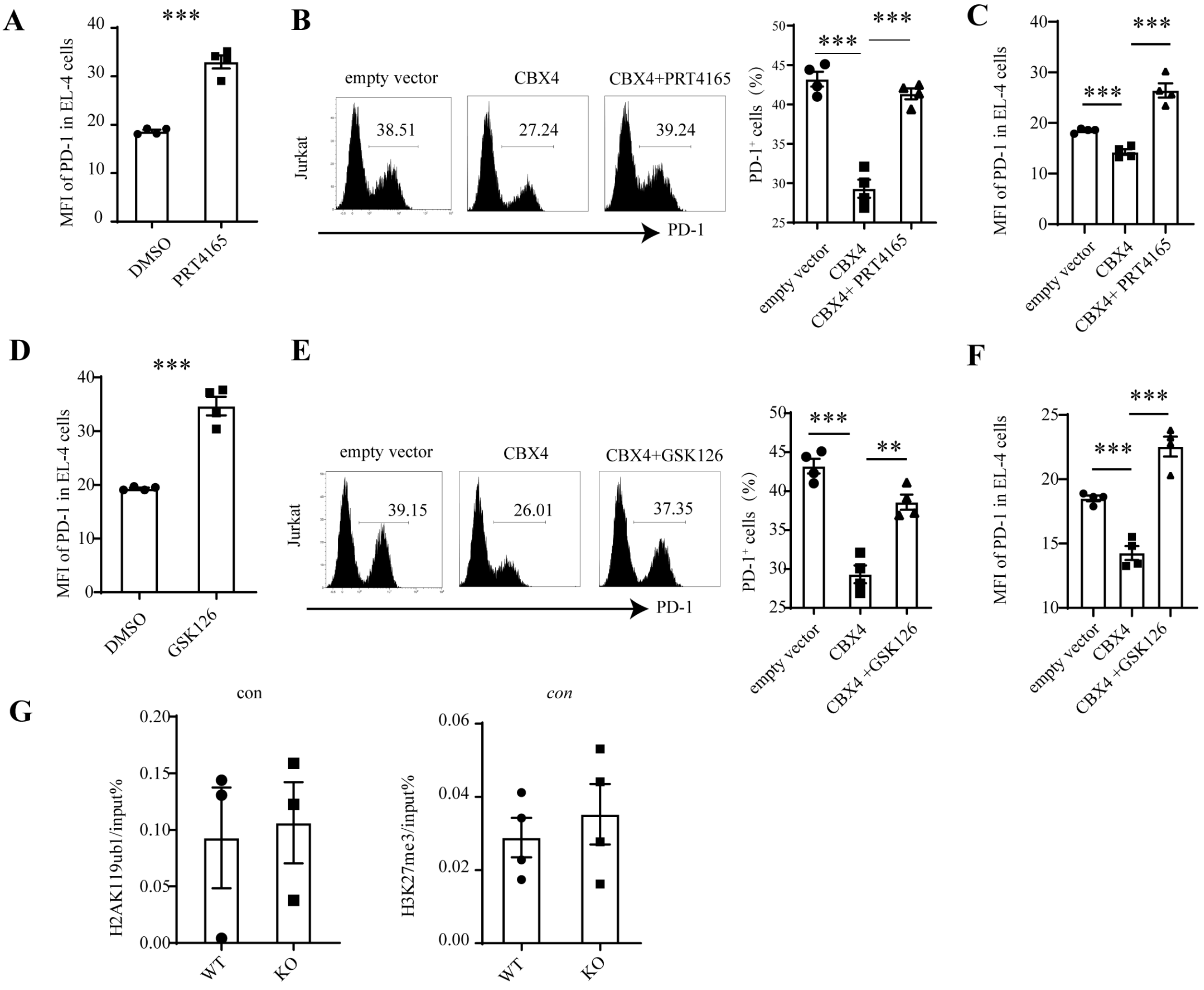

Supplementary Figure S6
