# Supplementary material for: CBX4 promotes antitumor immunity by suppressing Pdcd1 expression in T cells
Source: Mol Oncol. 2023 Oct 9;17(12):2694–708. doi: 10.1002/1878-0261.13516 (PMC10701776; doi:10.1002/1878-0261.13516)
Supplement: Supplementary file 1 — Table S1. Primer sequences for RT‐PCR. Table S2. Primer sequences for ChIP‐qPCR. Fig. S1. (related to Fig. 1). Absence of Cbx4 did not affect T‐cell development. Fig. S2. (related to Fig. 1). LAG‐3 expression in tumor‐infiltrating CD4+ and CD8+ T cells from WT and Cbx4 KO mice. Fig. S3. (related to Fig. 2). Cbx4 deficiency had no significant impact on other negative regulators. Fig. S4. (related to Fig. 2). PD‐1+ cells were present at an increased frequency among Cbx4‐deficient T cells. Fig. S5. (related to Figs 3, 4). Cbx4‐deficient T cells exhibit activation defects. Fig. S6. (related to Fig. 5). CBX4 promotes the formation of inhibitory histone modifications at the Pdcd1 locus. [file MOL2-17-2694-s001.zip › mol213516-sup-0002-DataS1.docx]

**Supporting Information.**

**Supplementary Table S1. Primer sequences for RT-PCR**

**Supplementary Table S2. Primer sequences for ChIP-qPCR**

**Supplementary Figure S1 (related to Figure 1). The absence of *Cbx4* did not affect T cell development. (A)** Cbx4 protein expression in MACS-purified CD3^+^T cells, CD4^+^T cells and CD8^+^T cells from WT and *Cbx4* KO mice. β-actin served as a loading control. **(B)** Thymocytes from WT and KO mice were stained for CD4 and CD8. Representative dot plots are shown on the left. The number of double-negative (DN), double-positive (DP), CD4 single-positive (SP), and CD8 SP thymocytes are shown on the right as Mean ± SE. **(C)** Total number CD4^+^ and CD8^+^ cells in the spleen (SPL) and lymph node (LN).Statistical data are presented as Mean ± SE . Each experiment was repeated at least 3 times. Unpaired t-test was used for comparison.

**Supplementary Figure S2 (related to Figure 1). LAG-3 expression in tumor infiltrating CD4^+^ and CD8^+^T cells from WT and *Cbx4* KO mice.**  Each experiment was repeated at least 3 times. Data are shown as Mean ± SE. Unpaired t-test was used for comparison.

**Supplementary Figure S3 (related to Figure 2). *Cbx4* deficiency had no significant impact on other negative regulators. (A)** Gating strategies of CD4^+^ or CD8^+^ naive T cells, central memory T cells and effector memory T cells.**(B-C)** Relative mRNA expression for *Lag3, Ctla4, Havcr2, Egr2/3, Ikzf1 and Tle4* in sorted naïve CD4^+^ and CD8^+^ T cells **(B)** and T cells treated with anti-CD3 and anti-CD28 for 3h **(C),** as determined by quantitative RT-PCR. Statistical data are presented as Mean ± SE . Each experiment was repeated at least 3 times. Unpaired t-test was used for comparison.

**Supplementary Figure S4 (related to Figure 2). PD-1^+^ cells were present at an increased frequency among *Cbx4*-deficient T cells.** Spleen cells from WT and *Cbx4* KO mice were stained for the expression of various molecular markers. **(A&B)** PD-1 expression in CD4^+^ **(A)** and CD8^+^ **(B)** CD44^+^CD62L^+^effector memory (Tem) and CD44^+^CD62L^-^ central memory (Tcm)T cells. **(C&D)** TIM-3, LAG-3 and CTLA-4 expression in CD4^+^ **(C)** and CD8^+^ **(D)** T cells . **(E)** Percentage of CD25^+^Foxp3^+^ regulatory T cell in CD4^+^ T cells and PD-1 expression in regulatory T cells .Statistical data are presented as Mean ± SE. Each experiment was repeated at least 3 times.. Unpaired t-test was used for comparison. **P*< 0.05, ***P*< 0.01.

**Supplementary Figure S5 (related to Figure 3, 4). *Cbx4*-deficient T cells exhibits activation defects. (A)** Gating strategies of stimulated Jurkat, EL-4 cells. **(B)** Naïve CD4^+^T cells from WT and *Cbx4* KO mice were cultured under Th2, Th17 and iTreg polarizing conditions. The percentage of IL-4^+^, IL-17^+^, and CD25^+^Foxp3^+^ cells in the cultures was determined by flow cytometry. **(C)** Wild-type or *Cbx4* KO CD4^+^CD45RB^hi^ naïve T cells were adoptively transferred into *Rag1*^-/-^recipient mice. The ratios of CD4^+^ in CD45^+^ cells in peripheral blood of *Rag1*^-/-^recipient mice, which were examined by flow cytometry (n=3 for each group). **(D)** Colon length of *Rag1*^-/-^ mice with no colitis was recorded. Data from 3 independent experiments are presented as Mean ± SE. Unpaired t-test was used for comparison.

**Supplementary Figure S6 (related to Figure 5). CBX4 promotes the formation of inhibitory histone modifications at the *Pdcd1* locus.** **(A)** PD-1 expression on EL-4 cells stimulated with anti-CD3 and CD-28 for 24h with the addition of PRT4165 or DMSO. **(B)** Jurkat cells were transfected Myc-CBX4 (CBX4) or empty vector. The transfectants were treated with 1$\mu$M PRT4165 for 24h before being examined for PD-1 expression. **(C)** EL-4 cells were transfected with Flag-CBX4 or empty vectors., The transfectants were treated with 1$\mu$M PRT4165 for 24h before being examined for PD-1 expression.**(D)** PD-1 expression on EL-4 cells stimulated with anti-CD3 and CD-28 for 24h with the addition of GSK126 or DMSO. **(E)** Jurkat cells were transfected Myc-CBX4 (CBX4) or empty vector. The transfectants were treated with 1$\mu$M GSK126 for 24h before being examined for PD-1 expression. **(F)** EL-4 cells were transfected with Flag-CBX4 or empty vectors, The transfectants were treated with 1$\mu$M GSK126 for 24h before being examined for PD-1 expression**.(G)** H2AK119ub1 and H3K27me3 modification at *con* of the *Pdcd1* locus at 0.5h were analyzed by ChIP-qPCR. Data from at least 3 independent experiments are presented as Mean ± SE. Unpaired t-test was used for comparison. ***P*< 0.01, ****P*< 0.001.
